# Mental Well-Being Among Adversity-Exposed Adolescents During the COVID-19 Pandemic

**DOI:** 10.1001/jamanetworkopen.2024.2076

**Published:** 2024-03-13

**Authors:** Julia H. Raney, Shayna Weinstein, Kyle T. Ganson, Alexander Testa, Dylan B. Jackson, Matthew Pantell, David V. Glidden, Claire D. Brindis, Jason M. Nagata

**Affiliations:** 1 Division of Adolescent and Young Adult Medicine, Department of Pediatrics, University of California, San Francisco; 2 School of Health, University of Berkeley, Berkeley, California; 3 Factor-Inwentash Faculty of Social Work, University of Toronto, Toronto, Ontario, Canada; 4 Department of Management, Policy and Community Health, University of Texas Health Science Center at Houston, Houston; 5 Department of Population, Family and Reproductive Health, Johns Hopkins Bloomberg School of Public Health, Baltimore, Maryland; 6 Division of Hospital Medicine, Department of Pediatrics, University of California, San Francisco; 7 Department of Epidemiology and Biostatistics, University of California, San Francisco; 8 Philip R. Lee Institute for Health Policy Studies, University of California, San Francisco

## Abstract

**Importance:**

Further research is needed to understand factors associated with well-being during the COVID-19 pandemic among adolescents who have experienced adverse childhood experiences (ACEs).

**Objective:**

To explore factors associated with improved mental health during the COVID-19 pandemic among adolescents who have experienced ACEs.

**Design, Setting, and Participants:**

This cross-sectional study used data from the baseline (2016-2018) and sixth (March 2021) COVID Rapid Response Research (RRR) surveys of the Adolescent Brain Cognitive Development study, which includes 21 sites across the US. Adolescents aged 11 to 15 years who completed the COVID RRR mental health measures were included. Data analyses were conducted from June to August 2023.

**Exposures:**

School-based factors (eg, in-person school) and 8 coping behaviors (eg, exercise).

**Main Outcomes and Measures:**

The primary outcomes were adolescent-reported positive affect (PA) and perceived stress (PS). Adolescents were stratified by no ACEs, low-to-intermediate ACEs (1-3), and high ACEs (≥4). Linear regressions estimated associations between factors and mental health, adjusting for potential confounders. Unstandardized beta coefficients (B) were compared with equality of coefficients tests.

**Results:**

The 4515 adolescents in this study (mean [SD] age, 13.3 [0.88] years; 51% [95% CI, 50% to 53%] female) were racially and ethnically diverse (American Indian/Alaska Native, 2% [95% CI, 2% to 3%]; Asian, 8% [95% CI, 7% to 9%]; Black, 11% [95% CI, 10% to 12%]; Latino or Hispanic, 17% [95% CI, 15% to 18%]; White, 61% [95% CI, 60% to 63%]; other, 1% [95% CI, 0% to 2%]). For youths with high ACEs, caring for one’s body (PA B = 4.02 [95% CI, 1.39 to 6.66]; PS B = −0.92 [95% CI, −1.84 to 0.00]), exercising (PA B = 3.19 [95% CI, 0.46 to 5.92]; PS B = −1.41 [95% CI, −2.40 to −0.43]), and engaging in healthy behaviors (PA B = 4.07 [95% CI, 1.28 to 6.84]; PS B = −1.01 [95% CI, −1.98 to −0.05]) were associated with higher PA and lower PS scores. In-person schooling had a greater impact on PA scores for youths with high ACEs (B = 5.55 [95% CI, 2.08 to 9.01]) than youths with low-to-intermediate ACEs (B = 1.27 [95% CI, 0.27 to 2.27]).

**Conclusions and Relevance:**

These findings suggest that in-person schooling and several coping behaviors (caring for one’s body, exercising, and engaging in healthy behaviors) were associated with significantly higher PA and lower PS during the COVID-19 pandemic among adolescents with high ACEs. Adolescents with high ACEs demonstrated especially greater mental health scores when they reported in-person schooling. Future studies should build on these findings to identify clinical and school-based mental health protective factors for adolescents with high ACE risk.

## Key Points

**Question:**

What factors were associated with higher adolescent well-being and lower stress during the COVID-19 pandemic, particularly among adolescents who had adverse childhood experiences (ACEs)?

**Findings:**

In a national, cross-sectional study of 4515 adolescents, in-person schooling, caring for one’s body (eg, meditating), exercising, and engaging in healthy behaviors (eg, sleep) were associated with significantly higher positive affect and lower perceived stress scores among adolescents with ACEs.

**Meaning:**

These findings suggest that in-person schooling and several coping behaviors were associated with significantly higher positive affect and lower perceived stress during the COVID-19 pandemic among adolescents with high ACEs, and future studies should build on these findings to identify protective mental health factors for adolescents with high ACE risk.

## Teaser

This cross-sectional study explores factors associated with improved mental health during the COVID-19 pandemic among adolescents who had adverse childhood experiences.

## Introduction

Adolescent mental health is in crisis. Since March 2020, adolescent anxiety and depressive symptoms have doubled globally.^1^ The worsening trend in mental health among adolescents is partially secondary to the profound impact of COVID-19 on adolescents’ school and social routines.^2,3,4,5^ In 2021, among US adolescents, 41% shared that they persistently felt sad or hopeless and demonstrated higher rates of stress and anxiety.^2,6,7^ In addition, rates of suicidal ideation in adolescents increased from 17% in 2017 to 37% during the pandemic.^8^ Even as adolescents have reentered schools, rates of poor adolescent mental health remain unacceptably high, with substantial disparities by sex, race, and ethnicity.^2^ For example, a US cohort study found that Asian, Black, and multiracial adolescents reported greater COVID-19–related distress and discrimination than did non-Hispanic White adolescents.^9^

A critical risk factor for poor adolescent mental health is a history of adverse childhood experiences (ACEs), which are defined as potentially traumatic events (eg, experiences of neglect, abuse, and household dysfunction) that occur in childhood and adolescence.^10^ Prepandemic studies^11^ have found that ACEs are associated with a higher likelihood of all mental health diagnoses, particularly for youths exposed to 4 or more ACEs. Although any ACE exposure increases a person’s risk for numerous mental and physical health conditions, exposure to 4 or more ACEs places an individual at high risk for developing adverse health outcomes, ranging from asthma to obesity to neuropsychiatric disorders.^12,13,14^ This occurs because experiencing high levels of adversity, without adequate buffering, can cause excessive activation of the stress response, termed *toxic stress*, which can have cascading neurodevelopmental effects and is associated with higher rates of adolescent anxiety, depression, and behavioral problems.^11,15,16^ During the COVID-19 pandemic, those reporting 4 or more ACEs were at 4 times the risk of poor mental health and at 25 times the risk of past-year suicide attempts, compared with those without ACEs.^17^ Adversity-exposed youths may have been at higher risk of negative consequences than youths without ACEs because COVID-19 acted as a universal stressor, which may have had a higher likelihood of overwhelming the coping capacity of youths who were already approaching their maximum tolerance of stress.

As we continue to learn to cope and adapt to the current pandemic, it is critical to understand factors associated with improved adolescent mental health, especially among youths who have experienced ACEs. Prior national US studies^18,19^ assessing adolescent mental health during the pandemic have identified social connectedness, sleep, physical activity, and parental involvement as protective factors. In addition, virtual schooling has been widely implicated in the literature as a risk factor for adverse health outcomes among adolescents,^18,20,21^ although several studies report some benefits from virtual schooling (eg, increased sleep), suggesting that not all groups experienced similar consequences of online schooling.^22^ However, those studies did not focus on youths with high ACE scores, who have unique, intersecting risk factors and are at the highest risk for developing toxic stress.^11,23^ Studies examining adolescent mental health among adversity-exposed youths during the pandemic are limited. One study^24^ of Chinese college students found that participating in meditation mediated the positive association between ACE score and depression during the pandemic, but it did not explore additional factors. Identifying factors associated with improved adolescent mental health is a critical area of research to help promote health equity for our most vulnerable youths.

To help fill this gap, this study investigated factors associated with adolescent well-being, particularly among adolescents facing adversity, using a sociodemographically diverse, population-based sample of adolescents aged 11 to 15 years in the US. Factors were selected on the basis of prior literature and with a holistic approach that included multiple levels of each adolescent’s living conditions, including interpersonal relationships, learning environments, and individual behaviors that interact with and influence adolescent mental health and well-being.^25,26^ To our knowledge, this is the first study to investigate how schooling factors and coping behaviors are associated with mental health (ie, perceived stress [PS] and positive affect [PA]) 1 year into the pandemic for youths with high ACE scores.

## Methods

This study used a cross-sectional design to determine associations between school and coping factors and mental health among US early adolescents with high ACE scores during the COVID-19 pandemic. We analyzed survey data from the Adolescent Brain Cognitive Development (ABCD) study, which is the largest long-term study of brain development and child health in the US. The ABCD study includes participants from 21 sites across the country.^27^ During the COVID-19 pandemic, the ABCD study released 6 COVID Rapid Response Research (COVID RRR) surveys to both adolescents and parents, which provided additional data on coping behaviors, changes in schooling, and adolescent emotional health during this time from May 2020 to March 2021. This study uses adolescent responses to the March 2021 COVID RRR Survey 6 (the final COVID survey), when there was the greatest heterogeneity of in-person vs virtual schooling in our cohort (46% reported online schooling only). Adolescents who did not participate in either the PA or PS questionnaires for the COVID RRR Survey 6 were excluded (7447 adolescents) (eTable 1 in Supplement 1). Centralized institutional review board approval was received from the University of California, San Diego. Written informed consent was obtained from caregivers, and verbal assent was obtained from the children. This study followed the Strengthening the Reporting of Observational Studies in Epidemiology (STROBE) reporting guideline for cross-sectional studies.

### Exploratory Variables: School and Coping Factors

School and coping factors were measured by the COVID RRR Survey 6 on the basis of prior literature (see the eAppendix in Supplement 1 for further measure details).^18,19,25,26^ Adolescents reported schooling method and parental engagement with their schoolwork. Adolescents’ coping behaviors were assessed by yes and no questions to 8 actions (eg, made time to relax or exercising).

### Outcome Variables: Mental Health and Well-Being During the COVID-19 Pandemic

Adolescents’ mental health was measured by the COVID RRR Survey 6 (March 2021). We included 2 mental health measures to capture psychopathology (PS) and well-being (PA) according to the dual-factor mental health model, which has been used for young adolescents (eAppendix in Supplement 1).^28,29^ Scores for PS (range, 0-16) and PA (range,1-45) are continuous measures. A higher PS score indicates higher PS, and a higher PA score indicates higher well-being.^28,30^

### Interaction and Stratification Variables: ACEs

The ACE score, derived from 2016 to 2018 baseline surveys of the ABCD study, reflects the 10 ACEs from a Centers for Disease Control and Prevention and Kaiser Permanente study.^10^ Although the ABCD study did not use a validated ACEs screener, we followed a procedure similar to that of prior work^30,31,32^ to generate the ACEs variable. eTable 2 in Supplement 1 describes how ABCD survey questions map onto validated ACEs questions. ACE scores were classified into mutually exclusive categories (0, 1-3, and ≥4), aligning with established risk levels for toxic stress.^23^ Notably, an ACE score of 4 or higher indicates a higher risk, consistent with both prior research and our moderation analysis findings.^10,33^

### Covariates

Parents reported participants’ age, sex (male or female), race and ethnicity (American Indian/Alaska Native, Asian, Black, Latino or Hispanic, White, or other [ie, any other race not otherwise specified]), highest caregiver education (high school or lower vs college or higher), and annual household income (<$25 000, $25 000 to <$50 000, $50 000 to <$75 000, $75 000 to <$100 000, $100 000 to $200 000, and >$200 000) at baseline. Race and ethnicity were included to account for racial and ethnic variations in ACEs exposure and COVID-19–related stressors.^34,35,36^ Prepandemic mental health measures (externalizing and internalizing behaviors) were generated from parent and/or caregiver responses to the Child Behavior Checklist at the 1-year follow-up,^37^ just before the start of the pandemic (2018-2019). See the eAppendix in Supplement 1.

### Statistical Analysis

Data analyses were conducted from June to August 2023. We first stratified our data by the adolescents’ number of ACEs (0, 1-3, and ≥4). We used χ^2^ and analysis of variance tests to examine statistically significant differences between stratified proportions for categorical variables and numeric variables, respectively. We stratified the model according to the degree of ACE exposure and individually analyzed the impact of each school and coping factor on PA and PS using linear regressions, adjusting for confounders. We also calculated unstandardized beta coefficients (B) and standardized β coefficients. To assess whether the associations of these factors significantly varied across models stratified by ACE exposure, we used an equality of coefficients test.^38,39^ We used this approach instead of multiplicative interaction terms to simplify the interpretation of the results and to reduce the complexity introduced by a large number of interaction coefficients, because the study includes 10 independent variables across 2 dependent variables. All tests were considered significant at the *P* < .05 level (2-tailed). Missing data were handled through listwise deletion. All analyses included propensity weights based on the American Community Survey.^40^

## Results

The analytic sample of 4515 adolescents (mean [SD] age, 13.3 [0.88] years; 51% [95% CI, 50%-53%] female) was racially and ethnically diverse (American Indian/Alaska Native, 2% [95% CI, 2%-3%]; Asian, 8% [95% CI, 7%-9%]; Black, 11% [95% CI, 10%-12%]; Latino or Hispanic, 17% [95% CI, 15%-18%]; White, 61% [95% CI, 60%-63%]; other, 1% [95% CI, 0%-2%]) (Table 1). Table 2 shows results from the adjusted linear regression models examining cross-sectional associations between mental health and well-being measures (PA and PS), stratified by adolescents with 0 ACEs, 1 to 3 ACEs, and 4 or more ACEs. eTable 3 in Supplement 1 shows standardized β coefficients.

**Table 1.** Sociodemographic Characteristics, School and Coping Factors, Pandemic Mental Health, and ACEs of Participants in the Adolescent Brain Cognitive Development Study

Characteristics	Participants, % (95% CI)^a^				*P* value
	Total (N = 4515)	0 ACEs (n = 1159)	1-3 ACEs (n = 3124)	≥4 ACEs (n = 232)	
Demographic characteristics					
Age, mean (SD), y	13.3 (0.88)	13.3 (0.89)	13.3 (0.89)	13.3 (0.85)	.57
Sex					
Female	51 (50-53)	53 (50-56)	51 (49-53)	52 (45-59)	.56
Male	49 (47-50)	47 (44-50)	49 (47-51)	48 (41-55)	
Race and ethnicity					
American Indian/Alaska Native	2 (2-3)	1 (1-2)	2 (1-3)	4 (2-8)	<.001^c^
Asian	8 (7-9)	15 (13-18)	6 (5-7)	2 (1-6)	
Black	11 (10-12)	8 (7-10)	11 (10-12)	15 (11-19)	
Latino or Hispanic	17 (15-18)	18 (15-20)	17 (15-18)	13 (9-19)	
White	61 (60-63)	57 (53-60)	63 (61-64)	65 (58-71)	
Other^b^	1 (0-2)	1 (1-3)	1 (1-2)	1 (0-6)	
Highest parent education					
College education or more	87 (86-88)	89 (87-91)	87 (85-88)	85 (79-89)	.22
High school education or less	13 (12-14)	11 (9-13)	13 (12-15)	15 (11-21)	
Annual household income, $					
<25 000	11 (10-13)	7 (5-9)	11 (10-13)	19 (14-25)	<.001^c^
25 000 to <50 000	17 (16-19)	15 (12-18)	17 (15-19)	29 (22-35)	
50 000 to <75 000	18 (17-20)	16 (13-19)	18 (16-19)	33 (26-40)	
75 000 to <100 000	19 (17-20)	20 (17-23)	19 (18-21)	11 (7-16)	
100 00 to 200 000	27 (26-28)	30 (28-34)	28 (26-29)	7 (5-11)	
>200 000	8 (7-9)	12 (10-14)	7 (7-8)	1 (0-2)	
Prepandemic mental health measures^d^					
Internalizing problems	9 (7-10)	5 (3-6)	9 (8-10)	18 (13-24)	<.001^c^
Externalizing problems	4 (3-5)	1 (0-2)	4 (3-5)	14 (10-20)	<.001^c^
School and coping factors					
Schooling					
Online only	46 (44-48)	49 (46-53)	46 (44-48)	40 (34-47)	.13
In-person	28 (26-29)	24 (21-27)	29 (27-31)	30 (24-37)	
Hybrid	22 (21-24)	23 (20-26)	22 (20-23)	26 (20-33)	
Other	3 (3-4)	4 (2-5)	3 (27-42)	4 (2-7)	
Parental involvement with schoolwork, mean (SD), d/wk	2.2 (2.4)	1.9 (2.3)	2.2 (2.4)	2.6 (2.5)	.01
Coping behaviors (past week)					
Took breaks from news	48 (46-50)	51 (47-54)	47 (45-49)	50 (43-57)	.47
Neighborhood social distance activity (eg, decorated windows or driveway)	7 (62-77)	7 (56-89)	5 (3-9)	7 (6-8)	.09
Took care of body (eg, meditating, stretching, or deep breathing)	45 (43-47)	47 (44-51)	45 (43-47)	39 (32-46)	.03
Exercise (eg, walking, running, or an online exercise class)	61 (59-62)	64 (60-67)	61 (59-62)	54 (47-60)	.01
Spent more time on hobbies or started a new one	49 (47-51)	50 (47-53)	50 (48-52)	39 (32-46)	.004
Engaged in healthy behaviors (eg, trying to eat healthy or getting plenty of sleep)	39 (38-41)	42 (38-45)	39 (37-41)	31 (24-37)	.01
Made time to relax	53 (52-55)	54 (50-57)	54 (52-56)	48 (40-55)	.22
Connected with others online or by phone	61 (60-63)	63 (60-66)	61 (59-63)	58 (50-64)	.30
Mental health and well-being outcomes					
Positive affect score, mean (SD)	31.9 (9.4)	32.5 (9.2)	31.9 (9.2)	30.5 (9.8)	.02
Total perceived stress score, mean (SD)	5.7 (3.1)	5.3 (3.0)	5.7 (3.0)	6.6 (3.4)	<.001^c^
No. of self-reported ACEs					
0	25 (24-26)	NA	NA	NA	NA
1-3	68 (67-70)	NA	NA	NA	NA
≥4	7 (6-8)	NA	NA	NA	NA

Abbreviations: ACE, adverse childhood experience; NA, not applicable.

^a^ Propensity weights from the Adolescent Brain Cognitive Development Study were applied on the basis of the American Community Survey from the US Census.

^b^ Other refers to any race or ethnicity not better defined by American Indian/Alaska Native, Asian, Black, Latino or Hispanic, or White.

^c^ *P* value was calculated with χ^2^ and analysis of variance tests.

^d^ Year 1 follow-up (2018-2020) was for prepandemic mental health measures.

**Table 2.** Associations of School and Coping Factors With Mental Health Among 4515 Adolescents During the COVID-19 Pandemic

Factors	B (95% CI)^a^					
	Positive affect			Perceived stress		
	0 ACEs	1-3 ACEs	≥4 ACEs	0 ACEs	1-3 ACEs	≥4 ACEs
Schooling						
Online only	0 [Reference]	0 [Reference]	0 [Reference]	0 [Reference]	0 [Reference]	0 [Reference]
In-person	1.88 (0.06 to 3.69)^b^	1.27 (0.27 to 2.27)^b^^,^^c^	5.55 (2.08 to 9.01)^d^	−0.20 (−0.75 to 0.36)	−0.50 (−0.83 to −0.17)^d^	−1.48 (−2.69 to −0.28)^b^
Hybrid	0.87 (−0.96 to 2.71)	1.31 (0.27 to 2.34)^b^	0.60 (−2.89 to 4.08)	0.11 (−0.44 to 0.67)	−0.18 (−0.52 to 0.15)	−1.06 (−2.23 to 0.11)
Other^e^	−3.24 (−8.35 to 1.87)	−0.11 (−2.27 to 2.05)	2.45 (−5.21 to 10.10)	0.20 (−0.92 to 1.32)	−0.17 (−0.87 to 0.52)	−0.92 (−4.41 to 2.57)
Parental involvement with schoolwork, No. of d/wk	0.23 (−0.06 to 0.52)^c^	0.34 (0.18 to 0.51)^b^	0.89 (0.36 to 1.42)^d^	−0.04 (−0.13,0.05)	−0.05 (−0.10 to −0.01)^b^	−0.13 (−0.31 to 0.05)
Coping behaviors (past week)						
Took breaks from news	1.71 (0.44 to 2.96)^d^	1.78 (1.07 to 2.49)^f^	2.17 (−0.55 to 4.90)	−0.45 (−0.83 to −0.07)^b^	−0.27 to −0.51 to −0.04)^b^	−0.78 (−1.69 to 0.13)
Neighborhood social distance activity (eg, decorated windows or driveway)	1.43 (−1.54 to 4.41)	3.19 (1.89 to 4.50)^f^	2.13 (−3.45 to 7.71)	−0.04 (−0.71 to 0.62)^c^	−0.40 (−0.80 to 0.00)^b^^,^^c^	−2.29 (−3.98 to −0.60)^d^
Took care of body (eg, meditating, stretching, or deep breathing)	2.42 (1.25 to 3.79)^f^	2.83 (2.11 to 3.56)^f^	4.02 (1.39 to 6.66)^d^	−0.50 (−0.89 to −0.12)^b^	−0.46 (−0.69 to −0.22)^f^	−0.92 (−1.84 to 0.00)^b^
Exercised (eg, walking, running, or an online exercise class)	2.82 (1.52 to 4.13)^f^	2.60 (1.85 to 3.36)^f^	3.19 (0.46 to 5.92)^b^	−1.07 (−1.47 to −0.67)^f^	−0.60 (−0.84 to −0.35)^f^	−1.41 (−2.40 to −0.43)^d^
Spent more time on hobbies or started a new one	1.30 (0.06 to 2.54)^b^	2.01 (1.29 to 2.73)^f^	0.34 (−2.55 to 3.24)	−0.41 (−0.80 to −0.04)^b^	−0.05 (−0.29 to 0.19)	−0.02 (−1.01 to 0.98)
Engaged in healthy behaviors (eg, trying to eat healthy or getting plenty of sleep)	3.17 (1.95 to 4.40)^f^	3.68 (2.96 to 4.40)^f^	4.07 (1.28 to 6.84)^d^	−1.18 (−1.56 to −0.81)^f^	−0.84 (−1.08 to −0.60)^f^	−1.01 (−1.98 to −0.05)^b^
Made time to relax	1.65 (0.41 to 2.90)^d^	3.42 (2.71 to 4.12)^f^	2.37 (−0.36 to 5.09)	−0.88 (−1.26 to −0.50)^f^	−0.96 (−1.18 to −0.73)^f^	−0.45 (−1.38 to 0.48)
Connected with others online or by phone	−0.24 (−1.56 to 1.10)	0.22 (−0.54 to 0.98)	0.69 (−2.03 to 3.40)	0.11 (−0.29 to 0.51)	0.59 (−0.18 to 0.30)	0.21 (−0.67 to 1.08)

Abbreviation: ACE, adverse childhood experience.

^a^ Adolescent Brain Cognitive Development Study propensity weights were applied on the basis of the American Community Survey from the US Census. We used separate models for each factor of interest. Models were adjusted for sex, age, race and ethnicity, annual household income, parent education, site, and prepandemic mental health. B refers to unstandardized beta coefficient.

^b^ *P* < .05.

^c^ Indicates significant difference in coefficients with 4 or more ACEs as a reference from an equality of coefficient test.

^d^ *P* < .01.

^e^ Other may be defined as a school modality not better defined by online only, in-person, or hybrid.

^f^ *P* < .001.

### School Factors

In-person schooling had a statistically significantly greater impact on PA scores for youths with high ACEs (B = 5.55 [95% CI, 2.08 to 9.01]) than youths with low-to-intermediate ACEs (B = 1.27 [95% CI, 0.27 to 2.27]). In-person schooling was also associated with lower PS scores for adolescents with 4 or more ACEs (B = −1.48 [95% CI, −2.69 to −0.28]). Parental involvement with schoolwork was also associated with statistically significantly higher PA scores for adolescents with 4 or more ACEs (B = 0.89 [95% CI, 0.36 to 1.42]) compared with adolescents with 0 ACEs (B = 0.23 [95% CI, −0.06 to 0.52]).

### Coping Factors

For adolescents with 4 or more ACEs, taking care of one’s body (PA B = 4.02 [95% CI, 1.39 to 6.66]; PS B = −0.92 [95% CI, −1.84 to 0.00]), exercising (PA B = 3.19 [95% CI, 0.46 to 5.92]; PS B = −1.41 [95% CI, −2.40 to −0.43]), and engaging in healthy behaviors (PA B = 4.07 [95% CI, 1.28 to 6.84]; PS B = −1.01 [95% CI, −1.98 to −0.05]) were associated with higher PA and lower PS scores. In addition, neighborhood social distance activity was associated with a lower PS score in adolescents with ACEs 4 or more ACEs (B = −2.29 [95% CI, −3.98 to −0.60]) than those with 1 to 3 ACEs (*B* = −0.40 [95% CI, −0.80 to 0.00]) or no ACEs (B = −0.04 [95% CI, −0.71 to 0.62]).

## Discussion

In this cross-sectional study of a demographically diverse sample of US adolescents aged 11 to 15 years, we identified several factors associated with improved adolescent mental health and well-being among youths who have experienced ACEs. Among adolescents who had experienced high ACEs, in-person schooling, taking care of the body (stretching, meditating, and deep breathing), exercise, and engaging in healthy behaviors (eating healthy and sleeping well) were all associated with higher PA and lower PS scores. This is consistent with prior pandemic literature^24^ that found that meditation is associated with fewer depressive symptoms among adversity-exposed youths. In addition, national studies^18,19,20^ of adolescent health have also determined that physical activity, in-person school, and family connectedness are associated with improved mental health.

We believe the clinical importance of these associations to be substantial for several reasons. First, prior literature^29,41^ has used the PA and PS scales similarly to assess adolescent mental health correlates. Furthermore, given that stress and well-being are of critical importance in adolescent development and that adolescents experience ACEs at unacceptably high rates, we believe these associations will have major impacts on adolescent mental health at a population level.

In addition, we found that several coping factors in particular were significantly associated with greater well-being among youths exposed to high ACEs. For example, in-person schooling was associated with more significant improvements in PA among adolescents with high ACEs compared with youths with 1 to 3 ACEs, with an approximately 4-point higher PA score. This is analogous to an adolescent reporting, for example, that, instead of somewhat true, they think it is very true that they felt energetic and confident in the past week. This builds on the findings of Hertz et al^20^ of the positive associations between adolescent mental health and in-person schooling by demonstrating that in-person schooling is particularly important among youths with high ACE scores.

In addition, participating in a neighborhood social distance activity was more associated with lower PS scores for adolescents with 4 or more ACEs than youths with few or no ACEs, with approximately 2-point lower PS scores among youths with high ACEs compared with those with no ACEs. This is analogous to, for example, an adolescent reporting that, instead of very often, they only sometimes felt unable to control important things in their life. The modest improvement for adolescents with ACEs is consistent with prior literature^42^ that highlights the importance of participating in community for adolescents who have experienced high ACEs.

We also found that connecting with peers online was the most widely used coping behavior but was not associated with significant mental health improvements for any group of adolescents. This contrasts with the significant associations observed with 2 other social interaction measures: in-person schooling and parental support with schoolwork. We hypothesize this may be secondary to the negative consequences of excessive screen time and social media use. Prior studies^43^ have demonstrated that adolescent screen time doubled and social media use greatly increased during the pandemic. Excessive screen time and social media use have been associated with anxiety, depression, and stress in adolescents, although some studies^43,44^ have noted potentially positive effects of a greater sense of connection. We suspect that online methods of communication were insufficient to replace in-person connections, which are typically observed in traditional schooling and parental support with schoolwork.

These findings establish an important foundation of research with potential future policy and clinical implications. First, adolescent-specific recommendations to conduct screenings of positive or protective elements in the context of great societal upheaval are limited. Furthermore, uncertainty of what to do for a positive ACE score for adolescents has been cited as a key barrier to ACEs screening by pediatric practitioners, and adolescent-specific recommendations are limited.^45,46^ Our findings help fill this gap by demonstrating that several factors were associated with meaningful improvements in mental health improvements among adolescents with high exposure to ACEs. This finding emphasizes the need to conduct prospective studies to establish causality so that protective factors may be incorporated into ACEs screenings to better provide targeted clinical, school, and policy recommendations during times of crisis.^42^

We recommend that studies continue to explore factors associated with well-being for youths with high ACE scores, as well as those with low-to-intermediate ACE scores, in both times of crisis and stability. In addition, Table 1 highlights that there were several statistically significant sociodemographic differences across high-adversity and low-adversity groups. Importantly, we did adjust for these covariates in our models, so we do not believe these differences explain the noted differences in our final model. However, this study does not explore differences in the impact of school and coping factors for youths with intersecting marginalized identities. Prior studies^47,48^ have shown that sex, race, and ethnicity moderate the associations between ACEs and adverse mental health outcomes. We recommend that future studies focus on these subpopulations and evaluate how race, ethnicity, sex, and lesbian, gay, bisexual, transgender, and queer identity moderate findings.

Of note, our findings highlight factors associated with adolescent mental health 1 year into the COVID-19 pandemic. At that time, adolescents’ lives were still greatly influenced by societal upheavals, with approximately one-half of the study’s participants attending school virtually. However, in March 2021, hospitalization rates were greatly decreased from the peak in December 2020 to January 2021,^49^ and the adult population was starting to have access to vaccinations (although no adolescent participants were yet eligible to receive the COVID-19 vaccine).^50,51^ Accordingly, these findings should be interpreted within the time period that the data were collected and not extrapolated to other periods of the COVID-19 pandemic.

### Limitations

This study has several important limitations. First, the study is cross-sectional and observational, so we cannot infer causality. We recommend that future studies explore factors longitudinally for youths experiencing high ACE scores. Second, the time points of the exploratory and dependent variables vary slightly; the exploratory variables include the past week’s schooling modality and coping behaviors, whereas the outcome mental health variables assess the past week’s PA and past month’s PS. This may partially explain why several factors have larger coefficients for PA measures than PS. In addition, there are no established clinical associations for PA or PS scores, so clinical importance must be extrapolated. We recommend that future research clearly establish clinically meaningful effects for these scales so that results may be efficiently translated to inform guidelines. In addition, the ACE screener is not validated, although eTable 2 in Supplement 1 highlights how the ABCD questions map onto validated ACE screeners. It is also important to consider that there were few adolescents with ACE scores of 4 or more, so we believe some of our null findings may be secondary to insufficient power from small cell sizes among those with high ACE exposure. In addition, our ACE score combines parent and adolescent reports, which may result in underreporting by caregivers^52^ and either overreporting or underreporting by adolescents.^53^ The approach used in this study captures both perspectives in an effort to minimize underreporting. In addition, our sample had fewer male, Black, Latino, and American Indian/Alaska Native participants, as well as individuals with lower household income and parental education levels, compared with the excluded population from the ABCD study. This may limit generalizability.

## Conclusions

In this cross-sectional study of a diverse cohort of 4515 US adolescents, in-person schooling and several coping behaviors (caring for one’s body, exercising, and engaging in healthy behaviors) were associated with higher PA and lower PS scores among adolescents with high ACEs during the COVID-19 pandemic. Adolescents with high ACE scores demonstrated especially improved mental health scores when they reported in-person schooling. As we prepare for potential future crises, we recommend that future studies build on these findings so that clinic and policy guidelines, as well as parents and educators, may identify protective factors to promote health equity and improved mental health among these adolescents at high risk of poor outcomes.

## Notes

Supplement 1. **eTable 1.** Comparison of Included vs. Excluded Participants **eTable 2.** ABCD ACEs Survey Questions **eAppendix.** Supplemental Methods
eReferences
**eTable 3.** Associations Between School and Coping Factors and Mental Health Among Adolescents With 0 ACEs, 1-3 3 ACEs and ≥4 ACEs During the COVID-19 Pandemic (n=4,515)

Supplement 2.
Data Sharing Statement
