# Supplementary material for: Mental Well-Being Among Adversity-Exposed Adolescents During the COVID-19 Pandemic
Source: JAMA Netw Open. 2024 Mar 13;7(3):e242076. doi: 10.1001/jamanetworkopen.2024.2076 (PMC10938185; doi:10.1001/jamanetworkopen.2024.2076)
Supplement: Supplement 1. — eTable 1. Comparison of Included vs. Excluded Participants eTable 2. ABCD ACEs Survey Questions eAppendix. Supplemental Methods eReferences eTable 3. Associations Between School and Coping Factors and Mental Health Among Adolescents With 0 ACEs, 1-3 3 ACEs and ≥4 ACEs During the COVID-19 Pandemic (n=4,515) [file jamanetwopen-e242076-s001.pdf]

Supplemental Online Content

Raney JH, Weinstein S, Ganson KT, et al. Mental well-being among adversity-exposed adolescents during the COVID-19 pandemic. *JAMA Netw Open*. 2024;7(3):e242076.
doi:10.1001/jamanetworkopen.2024.2076

eTable 1. Comparison of Included vs. Excluded Participants

eTable 2. ABCD ACEs Survey Questions

eAppendix. Supplemental Methods

eReferences

eTable 3. Associations Between School and Coping Factors and Mental Health Among Adolescents With 0 ACEs, 1-3 3 ACEs and ≥4 ACEs During the COVID-19 Pandemic (n=4,515)

This supplemental material has been provided by the authors to give readers additional information about their work.

© 2024 Raney JH et al. *JAMA Netw Open.*

eTable 1. Comparison of Included vs. Excluded Participants

Sociodemographic characteristics	% (95% CI) or Mean (SD)		P*
	Included (n= 4,515)	Excluded (n= 7,447)	
**Age**	13.3 (0.9)	13.3 (0.9)	0.55
**Sex (%)**			0.0001
Female	51% (50%, 53%)	47% (46%, 49%)	
Male	49% (47%, 50%)	53% (51%, 54%)	
**Race/ethnicity (%)**			<0.001
Asian	8% (7%, 9%)	4% (3%, 5%)	
Black	11% (10%, 12%)	21% (20%, 22%)	
Latino / Hispanic	17% (15%, 18%)	22% (21%, 23%)	
Native American	2% (2%, 3%)	4% (3%, 4%)	
White	61% (60%, 63%)	47% (46%, 49%)	
Other[a]	1% (0%, 2%)	2% (0%, 2%)	
**Household income (%)**			<0.001
$24,999 or less	11% (10%, 12%)	22% (21%, 23%)	
$25,000 to $49,999	17% (16%, 18%)	23% (22%, 24%)	
$50,000 to $74,999	18% (13%, 15%)	18% (17%, 19%)	
$75,000 to $99,999	19% (17%, 20%)	14% (13%, 15%)	
$100,000 to $199,999	27% (26%, 28%)	17% (17%, 18%)	
$200,000 and greater	8% (7%, 9%)	6% (5%, 6%)	
**Parent's highest education**			<0.001
College education or more	87% (86%, 88%)	75% (74%, 76%)	
High school education or less	13% (12%, 14%)	25% (24%, 26%)	
**Pre-pandemic mental health measures***			
Internalizing problems	9% (8%, 10%)	11% (10%, 12%)	0.0001
Externalizing problems	4% (3%, 5%)	6% (6%, 7%)	<0.001
**ACE score**	1.6 (1.2)	1.4 (1.2)	<0.001

ABCD propensity weights were applied based on the American Community Survey from the US Census.
[a]Other: if race/ethnicity not better defined by Asian, Black, Latino/Hispanic, Native American, or White
*Calculated with Chi-Square and ANOVA tests

© 2024 Raney JH et al. *JAMA Netw Open.*

eTable 2. ABCD ACEs Survey Questions

ACE	File name	Question	Question	ABCD Assessment*
**Physical abuse**	ptsd01	ksads_ptsd_raw_762	Shot, stabbed, or beaten brutally by a grown up in the home	KSADS-5 PTSD Module – Parent
	ptsd01	ksads_ptsd_raw_763	Beaten to the point of having bruises by a grown up in the home	KSADS-5 PTSD Module – Parent
**Sexual abuse**	ptsd01	ksads_ptsd_raw_767	A grown up in the home touched your child in their privates, had your child touch their privates, or did other sexual things to your child	KSADS-5 PTSD Module – Parent
	ptsd01	ksads_ptsd_raw_768	An adult outside your family touched your child in their privates, had your child touch their privates or did other sexual things to your child	KSADS-5 PTSD Module - Parent
**Household violence**	ptsd01	ksads_ptsd_raw_766	Witness the grownups in the home push, shove or hit one another	KSADS-5 PTSD Module – Parent
	fes02	fam_enviro6	Family members sometimes hit each other	Environment Scale- Parent Report
	fes02	fam_enviro3	Family members sometimes get so angry they throw things	Environment Scale- Parent Report
	abcd_fes01	fes_youth_q6	Family members sometimes hit each other	Family Environment

© 2024 Raney JH et al. *JAMA Netw Open.*

				Scale – Youth Report
	abcd_fes01	fes_youth_q3	Family members sometimes get so angry they throw things	Family Environment Scale – Youth Report
**Substance abuse in the household**	fhxp102	famhx_4_p	Has any blood relative of your child ever had any problems due to alcohol such as: marital separation or divorce, laid off or fired from work, arrests or DUIs; alcohol harmed their health; in an alcohol treatment program; suspended or expelled from school 2 or more times; isolated self from family, caused arguments or were drunk a lot?**	Family History Assessment – Parent
**Household mental illness**	fhxp201	fam_history_13_yes_no	Has ANY blood relative of your child ever attempted or committed suicide?**	Demographics survey – Parent<br><br>(ABCD Family History Assessment Part 2)
	fhxp102	fam_history_6_yes_no	Has ANY blood relative of your child ever suffered from depression, that is, have they felt so low for a period of at least two weeks that they hardly ate or slept or couldn't work or do whatever they usually do?**	Demographics survey – Parent<br><br>(ABCD Family History Assessment Part 1)

© 2024 Raney JH et al. *JAMA Netw Open.*

**Divorce/separation**	pdem02	demo_prnt_marital_v2	Divorced/separated	ABCD Parent Demographics Survey
**Criminal household member**	fhxp201	fam_history_9_yes_no	Has ANY blood relative of your child been the kind of person who never holds a job for long, or gets into fights, or gets into trouble with the police from time to time, or had any trouble with the law as a child or an adult?	Family History Assessment – Parent
**Emotional neglect**	crpbi01	crpbi_parent4_y	First caregiver (caregiver participating in study/completing protocol). Believes in showing his/her love for me.	CRPBI Acceptance Subscale – Youth
**Physical neglect**	pmq01	parent_monitor_q1_y	How often do your parents/guardians know where you are?***	Parental Monitoring Survey
	pmq01	parent_monitor_q3_y	If you are at home when your parents or guardians are not, how often do you know how to get in touch with them?***	Parental Monitoring Survey
**Emotional abuse**	ptsd01	ksads_ptsd_raw_764_p	A non-family member threatened to kill your child	KSADS-5 PTSD Module – Parent
	ptsd01	ksads_ptsd_raw_765_p	A family member threatened to kill your child	KSADS-5 PTSD Module – Parent

*All ACEs data were determined through parent and adolescent responses in the baseline (2016-2018), one-year follow-up (2017-2019), and first half of two-year follow-up (2018-2019) surveys. A yes response to any of the following ten ACEs at any timepoint was counted as one-point.

**one-point given if blood relative was mother or father

***one-point if never/almost never

© 2024 Raney JH et al. *JAMA Netw Open.*

eAppendix. Supplemental Methods

Measures

Positive Affect:

Positive affect was assessed using the scale from the National Institutes of Health Toolbox Emotion Battery (NIHTB-EB) for Children.[1] Nine questions were asked about how adolescents felt in the past week with the following question stem: "Please rate how each item describes you now or within the past week:"

1. "I felt attentive (that is, alert or able to pay attention)."
2. "I felt delighted."
3. "I felt calm."
4. "I felt at ease (Definition: relaxed, comfortable)."
5. "I felt enthusiastic (Definition: very excited)."
6. "I felt interested."
7. "I felt confident."
8. "I felt energetic."
9. " I felt able to concentrate."

Youth responses included: 1 = "Not true"; 3 = "Somewhat true"; 5 = "Very true." The questions assess both activated (i.e., happiness, joy) and unactivated (i.e., serenity, peace) aspects of positive affect. We calculated a sum score for each participant. Higher scores are indicative of a more positive affect.

Perceived Stress:

Four questions were asked to participants regarding perceived stress from the Perceived Stress Scale: [2]

1. "In the last month, how often have you felt confident about your ability to handle your personal problems?"
2. "In the last month, how often have you felt difficulties were piling up so high that you could not overcome them?"
3. "In the last month, how often have you felt that you were unable to control the important things in your life?"
4. "In the last month, how often have you felt that things were going your way?"

Youth responses included: 0 = "never"; 1 = "almost never"; 2 = "sometimes"; 3 = "fairly often"; 4 = "very often." The two positive behaviors (Questions 1 [Confidence] and 4 [Have it your way]) were reverse coded. Higher scores are correlated to more stress, scores were summed on the PSS-4.

Protective Factors:

Schooling:

School format was measured with one question that asked adolescents to report what method of schooling they participated in the past week.

1. “In the past week, was your schooling taking place:”

Youth responses included: 1 =” Online/school-at-home”; 2 = “In person” ; 3 = “In person some days and online some other days”; 4 = “Other.” We refer to option 3 “In person some days and online some other days” as “hybrid.”

Parent engagement with schoolwork was measured by one adolescent-reported measure.

2. “In the past week, about how many days per week was a parent (or other adult taking care of you) involved with your school work?”

Youth responses were reported on a scale of number of days per week: 0; 1; 2 ; 3; 4; 5; 6; 7.

Coping Behaviors:

Nine coping behaviors options were given to participants, with the following question stem: “And finally, in the past week, to cope, have you done any of the following?:”

1. “Took breaks from watching, reading, or listening to news stories, including social media”
2. “Participated in a neighborhood social distance activity (such decorated windows or driveway, singing from balcony or porch)”
3. “Took care of your body, such as taking deep breaths, stretching, or meditating”
4. “Exercised (such as walking, running, or an online exercise class)”
5. “Spent more time on hobbies, or started a new one”
6. “Engaged in healthy behaviors, like trying to eat healthy, getting plenty of sleep”
7. “Made time to relax”
8. “Connected with others online or by phone”
9. “None of the above”

Responses included: 1 = “Yes” and 2 = “No.” Responses for options 1-8 were included. Responses for option 9 were excluded.

Pre-pandemic mental health measures:

© 2024 Raney JH et al. *JAMA Netw Open.*

Adolescents' mental health was evaluated by The Child Behavior Checklist (CBCL), which is a component of the Achenbach System of Empirically Based Assessment (ASEBA). This screening tool asks a parent/caretaker about multiple psychiatric symptoms and behavior problems in children ages 4–18.[3, 4]

The CBCL consists is made up of eight syndrome scales:[5]

1. Anxious/depressed
2. Withdrawn/Depressed
3. Somatic complaints
4. Social problems
5. Thought problems
6. Attention problems
7. Rule-breaking behavior
8. Aggressive behavior

These group into two higher-order factors—internalizing and externalizing.[5]

Clinically meaningful internalizing (Anxious/Depressed, Withdrawn/Depressed, and Somatic Complaints) and externalizing disorders (Rule-Breaking Behavior and Aggressive Behavior) were determined through T scores.[6] Broadband scores were age-normed into t-scores (mean 50, standard deviation of 10). Scores 65-69 are considered borderline for a clinically significant behavioral problem and scores of 70 or higher are indicative or a clinical-range behavioral disorder. We examined t scores of internalizing and externalizing problems as 65 or higher.[7,8]

eReferences

1. Paolillo EW, McKenna BS, Nowinski CJ, Thomas ML, Malcarne VL, Heaton RK. NIH Toolbox® Emotion Batteries for Children: Factor-Based Composites and Norms. *Assessment*. Published online April 1, 2018:1073191118766396. doi:10.1177/1073191118766396

2. Cohen S, Kamarck T, Mermelstein R. A global measure of perceived stress. *Journal of Health and Social Behavior*. 1983;24(4):385-396. doi:10.2307/2136404

3. Achenbach TM, Ruffle TM. The Child Behavior Checklist and Related Forms for Assessing Behavioral/Emotional Problems and Competencies. *Pediatrics In Review*. 2000;21(8):265-271. doi:10.1542/pir.21-8-265

4. Barch DM, Albaugh MD, Avenevoli S, et al. Demographic, physical and mental health assessments in the adolescent brain and cognitive development study: Rationale and description. *Developmental Cognitive Neuroscience*. 2018;32:55-66. doi:10.1016/j.dcn.2017.10.010

5. American Psychological Association. Child Behavior Checklist (CBCL). https://www.apa.org/depression-guideline/child-behavior-checklist.pdf

6. Diler RS, Birmaher B, Axelson D, et al. The Child Behavior Checklist (CBCL) and the CBCL-Bipolar Phenotype Are Not Useful in Diagnosing Pediatric Bipolar Disorder. *J Child Adolesc Psychopharmacol*. 2009;19(1):23-30. doi:10.1089/cap.2008.067

7. Achenbach T, Rescorla L. Achenbach System of Empirically Based Assessment. In: Volkmar FR, ed. *Encyclopedia of Autism Spectrum Disorders*. Springer; 2013:31-39. doi:10.1007/978-1-4419-1698-3_219

8. Tang JT, Saadi A, Dunn EC, Choi K. Concordance in Child-Parent Reporting of Social Victimization Experiences in the Adolescent Brain Cognitive Development Study. *Academic Pediatrics*. 2023;23(4):747-754. doi:10.1016/j.acap.2022.09.018

© 2024 Raney JH et al. *JAMA Netw Open.*

eTable 3: Associations Between School and Coping Factors and Mental Health Among Adolescents With 0 ACEs, 1-3 3 ACEs and ≥4 ACEs During the COVID-19 Pandemic (n=4,515)

	**Positive Affect [PA]**			**Perceived Stress [PS]**		
	0 ACEs	1-3 ACEs	≥4 ACEs	0 ACEs	1-3 ACEs	≥4 ACEs
**School and Coping Factors**[a]	β	β	β	β	β	β
**Schooling**						
Online only	reference	reference	reference	reference	reference	reference
In-person	0.129	0.064	0.225	-0.052	-0.078	-0.192
Hybrid	0.065	0.058	0.060	0.005	-0.044	-0.159
Other[b]	-0.034	-0.002	0.026	0.006	-0.009	-0.023
Parental involvement with schoolwork (days per week)	0.079	0.077	0.226	-0.034	-0.037	-0.082
**Coping behaviors (past week)**						
Took breaks from news	0.109	0.100	0.109	-0.054	-0.033	-0.058
Neighborhood social distance activity (ie decorated windows or driveway)	0.076	0.100	0.027	-0.034	-0.028	-0.086
Took care of body (ie meditating, stretching, deep breadths)	0.141	0.153	0.218	-0.084*	-0.065	-0.117
Exercised (such as walking, running, or an online exercise class)	0.162	0.132	0.165	-0.163	-0.095	-0.125
Spent more time on hobbies or started a new one	0.073	0.108	0.042	-0.083	-0.010	-0.019
Engaged in healthy behaviors (ie trying to eat healthy, getting plenty of sleep)	0.150	0.194	0.194	-0.187	-0.116	-0.118

© 2024 Raney JH et al. *JAMA Netw Open.*

Made time to relax	0.091	0.187	0.143	-0.138	-0.152	-0.088
Connected with others online or by phone	-0.001	0.028	0.022	0.015	0.014	0.257

β indicates standardized beta coefficients
Model adjusts for sex, age, race/ethnicity, household income, parent education, site, pre-pandemic mental health. Standardized coefficients are not compatible with survey weights, so these were not applied.
[a]We used separate models for each factor of interest
[b]Other may be defined as a school modality not better defined by online only, in-person, or hybrid

© 2024 Raney JH et al. *JAMA Netw Open.*
