# Supplementary material for: Mental Well-Being Among Adversity-Exposed Adolescents During the COVID-19 Pandemic
Source: JAMA Netw Open. 2024 Mar 13;7(3):e242076. doi: 10.1001/jamanetworkopen.2024.2076 (PMC10938185; doi:10.1001/jamanetworkopen.2024.2076)
Supplement: Supplement 2. — Data Sharing Statement [file jamanetwopen-e242076-s002.pdf]

# Data Sharing Statement

Raney. Mental Well-Being Among Adversity-Exposed Adolescents During the COVID-19 Pandemic. *JAMA Netw Open*. Published March 13, 2024.
doi:10.1001/jamanetworkopen.2024.2076

**Data**
**Data available:** No

**Additional Information**
**Explanation for why data not available:** Data used in the preparation of this manuscript were obtained from the ABCD Study (https://abcdstudy.org), held in the NIMH Data Archive (NDA). Investigators may apply for data access through the NDA (https://nda.nih.gov/).
